# The Workroom

**Published:** 1908-06-27

**Authors:** 


### NURSING ADMINISTRATION.

### THE WORKROOM.

To most matrons uniforms are a source of perpetual worry. It is the nature of all people who have things provided for them, outside their own individual choice, to grumble, and by the same law of nature accidents which never or rarely assail private garments are always happening to the official garb. Uniform needs incessant watchfulness lest it shame alike the wearer and the provider, and the task of keeping her big family tidy is a serious addition to the matron's other duties. To have a well-equipped dressmaking department on the premises is the way out of the difficulty; it may almost be said that it is the only way. It must be frankly acknowledged that a badly equipped dressmaking department is a scourge to every one concerned with it, and that more money can be spent on ill-conducted enterprises of this nature than would suffice to buy well-fitting uniforms ready-made from a respectable firm. But the comfort and the true economy procured by having things done in the house under proper management are so striking that it is worth taking a good deal of trouble to establish this department on a thoroughly sound basis. A good workroom is the first essential to success. It should be well lighted and airy, and provided with good artificial lighting arrangements. A little practical architectural skill will often suffice to make excellent workrooms in the basement. The walls should be lined with presses, or broad shelves fitted with curtains to protect the contents from the dust. Some really good sewing machines, in proportion to the work to be undertaken, and proper appliances for cutting, fitting, pressing, etc., must be provided, and this outlay will bring in a rich return in economy and speed. There should be a small fitting room adjoining. The crucial difficulty is the sewing mistress, or head dressmaker. Upon the right choice of this important person the entire success of the department will depend. She must be a trained dressmaker, of proved character, endowed with the talent of controlling her subordinates, and instructing them in their work. Her work will differ in many important particulars from that to which the trade dressmaker is accustomed, and to attempt to fill such a post by advertisement would be to expose the matron to a bewildering choice of unsuitable candidates. In starting a new department of this description it would undoubtedly be a safer course to take some one who had filled a similar position, say, as second in command at a good hospital. When once the right person has been secured a succession of workers can be ensured by the usual hospital process of "training your own." A wise superintendent will consult her dressmaker as to the girls who are to work as apprentices, and will generally find that in this way suitable people for the work are brought to her notice. A good sewing mistress will command a salary of from £30 to £40 a year, living in, and having everything provided. Living out she would take at least £50, and would in that case probably receive in addition partial board. The apprentices would commence at 5s. a week, receiving partial board, dinner, and tea. They would rise to the position of assistant dressmakers at a salary of 15s. a week. Such a staff, the number of assistants and apprentices proportioned to the size of the hospital staff, ought to be able to undertake, in addition to all the ordinary needlework and mending in the establshment, all the uniforms, bonnets, cloaks aprons, caps, etc., a certain amount of upholstery, and possibly such articles as surgical bandages. But to be financially successful the department must adopt business methods. Contracts must be made once a year for the materials used in the workroom, and the greatest possible care employed in the selection of goods. There must be a good system of bookkeeping, and the exact cost of materials and wares must be balanced against the garments produced, so that the average cost of the uniform can be ascertained. There is no doubt that one main source of saving under such a system as we have sketched is in the skilled repairs. In the hands of a good dressmaker a garment which appears to be hopelessly spoilt may be renovated to look like new, and every such garment repaired counts for good in the total. Moreover, where there is a properly organised workroom, the mending of linen ought to be undertaken there week by week. It is inexcusable when the scanty leisure moments of nurses are confiscated to scramble through repairs and cobble up holes, which need good darns or sound patches. By the kindness of Miss Swift, matron of Guy's Hospital, we are able to give the exact cost of the uniforms in her workroom. The following articles are supplied annually as uniform at Guy's:—

*Sisters and nurses* (the sisters' dresses being of rather better quality)—

| | |
|---|---|
| 4 linen dresses. | 2 cloaks, summer and winter.* |
| 1 merino dress. | 6 aprons. |
| 6 caps. | 6 collars. |
| 1 bonnet. | Cape for wearing in corridors. |

* These do not want renewing every year.

*Servants*—

| | |
|---|---|
| 4 print dresses. | 6 caps. |
| 12 aprons (6 for scrubbing). | 6 collars. |

The cost per head of the department last year worked out as follows:—

| | |
|---|---|
| Goods supplied ... ... ... ... | £1,484 |
| Wages in workroom ... ... ... | 181 |
| Total cost ... ... ... ... | £1,665 |

When the number of persons provided with uniforms is reckoned out, the amazing result is that the cost per head of each set of garments is as follows:—

| | £ | s. | d. |
|---|---|---|---|
| Sisters ... ... ... ... ... | 2 | 0 | 4 |
| Nurses ... ... ... ... ... | 1 | 4 | 3 |
| Servants ... ... ... ... ... | 1 | 2 | 0 |

We think there can be no better argument in favour of having such work done on the premises than the publication of these figures.

